# Controversial Indications for Sentinel Lymph Node Biopsy in Breast Cancer Patients

**DOI:** 10.1155/2015/405949

**Published:** 2015-03-03

**Authors:** Hazem Assi, Eman Sbaity, Mahmoud Abdelsalam, Ali Shamseddine

**Affiliations:** ^1^Department of Internal Medicine, American University of Beirut Medical Center, Cairo Street, P.O. Box 11-0236, Riad El Solh, Beirut 1107 2020, Lebanon; ^2^Department of Surgery, American University of Beirut Medical Center, Cairo Street, P.O. Box 11-0236, Riad El Solh, Beirut 1107 2020, Lebanon; ^3^Department of Medicine, Dalhousie University, 6299 South Street, Halifax, NS, Canada B3H 4R2

## Abstract

Sentinel lymph node biopsy (SLNB) emerged in the 1990s as a new technique in the surgical management of the axilla for patients with early breast cancer, resulting in lower complication rates and better quality of life than axillary lymph node dissection (ALND). Today SLNB is firmly established in the armamentarium of clinicians treating breast cancer, but several questions remain. The goal of this paper is to review recent work addressing 4 questions that have been the subject of debate in the use of SLNB in the past few years: (a) What is the implication of finding micrometastases in the sentinel nodes? (b) Is ALND necessary in all patients who have a positive SLNB? (c) How accurate is SLNB after neoadjuvant therapy? (d) Can SLNB be used to stage the axilla in locally recurrent breast cancer following breast surgery with or without prior axillary surgery?

## 1. Introduction

The past century has witnessed dramatic changes in the management of breast cancer, from an era when modified radical mastectomy was the norm to an era when mastectomy or axillary lymph node dissection (ALND) is infrequently performed. Less extensive surgical treatments, which are accompanied by better quality of life, are becoming a viable option for many patients with breast cancer based on the extent of the disease and patient preference.

Historically, axillary lymph node dissection was the classical approach to the axilla in all breast cancer patients. In the late 1990s, leaders in breast surgery introduced sentinel lymph node biopsy to early breast cancer. In axillary lymph node dissection, the surgeon aims at removing all lymph nodes in the level I and II axilla. The concept of SLNB is to remove the first axillary lymph node or nodes to which the breast drains. Sentinel lymph node is done by injecting blue dye into the breast parenchyma intraoperatively. Then a skin incision is done at the axilla at the level of the hair line and the surgeon searches for and removes any blue appearing node. Another technique is by injecting radioactive colloid material in the breast and using a gamma probe to detect and remove all radioactive axillary lymph nodes. The SLNB can be done using either technique or doing both at the same time with a similar accuracy. Now SLNB is established as the standard of care in breast cancer with clinically node-negative axilla on presentation (Veronesi 2010, NSABP B-32). Axillary lymph node dissection carries a significant risk of complications, as demonstrated in many studies in the literature. A recent systematic review found the incidence of self-reported lymphedema after axillary dissection to be 28% [1]. Arm dysfunction and persistent pain are also common following axillary dissection, resulting in poor quality of life for those patients [2]. The importance of SLNB is to spare most patients the morbidities of dissecting all the axillary lymph nodes.

The NSABP (Landmark National Surgical Adjuvant Breast and Bowel Project) B04 randomized trial, which started in the 1970s, paved the way toward decreased reliance on axillary dissection. The early results of the trial indicated that patients with clinically node-negative breast cancer had similar survival outcomes whether they were treated with mastectomy and axillary dissection, total mastectomy with axillary radiation, or total mastectomy without axillary treatment. Similar survival outcomes were also seen for women with clinically node-positive disease who were treated with either radical mastectomy or total mastectomy with axillary radiation. A follow-up study published in 2002 indicated that these similarities in outcomes remained sustainable after 25 years [3].

These results do not mean that the axilla can be completely ignored in women with clinically negative axilla, as the importance of axillary staging is well established in determining prognosis of breast cancer cases and making treatment decisions. However, evidence emerged in the 1990s that, in patients with clinically node-negative axilla, the axilla could be managed more selectively with less anatomic disruption and less morbidity than that caused by axillary dissection [4]. The NSABP B-32 trial results published in 2010 showed that, in women with clinically negative axilla, sentinel lymph node biopsy (SLNB) provides survival benefits and regional control similar to those seen with axillary lymph node dissection (ALND) but with the advantage of fewer side effects [5]. The Axillary Lymphatic Mapping against Nodal Axillary Clearance (ALMANAC) trial found that women who underwent SLNB alone experienced less lymphedema and sensory deficit than women who underwent ALND. Women who underwent SLNB alone were also able to resume their normal daily activities more quickly than women who underwent ALND [6].

The current practice is to offer SLNB to breast cancer patients who present with clinically and radiologically negative axilla. If the sentinel lymph nodes were found to be involved then completion axillary dissection is done. However, this dogma has been challenged recently with the results from the Z0011 trial [7, 8]. Patients who present with a positive axilla are not candidates from SLNB and undergo ALND. Although sentinel node biopsy is now firmly established in the armamentarium of clinicians treating early breast cancer, several questions about its indications and implications remain. The goal of this paper is to review recent work addressing four questions that have been the subject of debate in the use of SLNB in the past few years: (a) What is the implication of finding micrometastases in the sentinel nodes? (b) Is ALND necessary in all patients who have a positive SLNB? (c) How accurate is SLNB after neoadjuvant therapy? (d) Can SLNB be used to stage the axilla in locally recurrent breast cancer following breast surgery with or without prior axillary surgery?

## 2. What Is the Implication of Finding Micrometastases in the Sentinel Nodes?

The prognostic value of micrometastases in the sentinel lymph nodes in breast cancer has also been the subject of discussion in recent years. A multicentre cohort study of women who underwent SLNB found that the presence of micrometastases in sentinel nodes did not affect overall survival or disease-free survival [9]. Similarly, a prospective study with 8 years of follow-up found no difference in overall or disease-free survival in breast cancer patients with sentinel node micrometasteses compared with those with negative sentinel nodes [10]. However, other studies have found that micrometastases in sentinel nodes are associated with poorer survival [11, 12]. More recent findings from the ACOSOG-Z0010 trial, which was designed to investigate the incidence and significance of micrometastases in sentinel lymph nodes and bone marrow in patients with early-stage breast cancer who underwent breast-conserving surgery and whole breast irradiation, suggest that micrometastases found only by immunohistochemistry are clinically insignificant [13]. Similarly, 10-year follow-up data from NASP B-32 presented at the 2013 San Antonio Breast Cancer Symposium indicated that the presence of occult metastatic disease in sentinel lymph nodes did not have a significant impact on survival or locoregional recurrence [14].

## 3. Is ALND Necessary in All Patients Who Have a Positive SLNB?

ASCO guidelines in 2005 recommended completion ALND to all patients with a positive sentinel lymph node. The results of the ACOSOG-Z0011 trial, which were published in 2010 and 2011, have led to a significant shift from the routine use of ALND in women with positive sentinel lymph nodes [15, 16]. In the Z0011 study, patients with early breast cancer with a positive SLNB who underwent lumpectomy and whole breast irradiation were randomly assigned to receive ALND or no further axillary dissection. SLNB plus ALND did not provide an additional survival benefit over SLNB alone, demonstrating that eligible breast cancer patients with positive sentinel lymph nodes could be spared an ALND without compromising survival [7, 8].

The IBCSG 23-01 trial began enrolling women 3 years after the Z0011 trial and further explored the need for ALND in women with positive sentinel lymph nodes. Although there were differences between the 2 trials in terms of eligibility criteria, the IBCSG 23-01 trial also randomly assigned participants to receive either ALND or no further axillary surgery. Most of the patients received radiation or chemotherapy or both. The 5-year disease-free survival was similar in the 2 groups [17].

The results of these 2 trials suggested that it is safe to omit axillary dissection in women with early breast cancer who have only 1 or 2 positive sentinel nodes. It should be noted, however, that all of the women in the Z0011 trial received whole breast irradiation, and a large proportion of the women in the IBCSG 23-01 trial also underwent radiation therapy. Given that level I and part of level II of the axilla are included in the treatment field with whole breast irradiation, women who received irradiation in the 2 trials probably experienced a therapeutic effect from radiation therapy sterilizing residual tumor, but it is not clear how much of a role this played in the trial outcomes.

The multicenter trial “After Mapping the Axilla: Radiotherapy or Surgery?” (AMAROS trial) was specifically designed to compare local and regional control and morbidity with axillary radiation therapy versus axillary surgery. Participants in this study had clinically node-negative disease but a positive sentinel lymph node. Results were presented at the 2013 Annual Meeting of the American Society of Clinical Oncology [18]. After 5 years of follow-up there were no significant survival differences between the groups who received axillary radiation and those who received axillary surgery. However, it has been argued that the low rates of axillary recurrence are an important limitation of the AMAROS trial results, because any differences in recurrence rates may have been undetectable [19]. Thus, ANLB cannot yet be replaced by axillary radiation as the standard of care in patients with a positive sentinel node.

Janni and colleagues predict that the role of ANLB in the treatment of patients with clinically normal axillae and positive sentinel lymph nodes will continue to decrease in importance [19]. Currently, for patients with micrometastasis on a sentinel lymph node we can confidently omit ALND, whether they undergo lumpectomy or mastectomy. SLNB positive patients that fit the Z0011 criteria are spared from ALND. This is reflected in the ASCO guidelines 2014 as opposed to the prior recommendations to dissect the axillae of all SLN positive patients. Z0011 data is based on partial mastectomy patients, so its results cannot be extrapolated to mastectomy patients. However, our practice is to offer those patients radiation instead of ALND when they fit the AMAROS trial eligibility criteria. In the meantime, it is helpful to discuss those cases in a multidisciplinary tumor board.

## 4. How Accurate Is SLNB after Neoadjuvant Chemotherapy?

According to the ASCO guidelines of 2005, SLNB is not accurate after NACT and thus cannot be offered to breast cancer patients undergoing preoperative chemotherapy. Chemotherapy is often administered before surgery in patients with stage II or III breast cancer, with large primary breast cancer tumors or lymph node involvement, with the aim of downsizing the size of the tumor so that less extensive surgery will be required. Clinical evidence of disease in the axillary lymph nodes often disappears following neoadjuvant therapy in patients who had initially presented with node-positive disease. It is important to determine whether any disease remains in the nodes after chemotherapy, but the optimal way to make this determination is the subject of debate. Do patients in this situation need to undergo complete dissection of the axilla, with its attendant complications, or can they safely be offered SLNB instead?

SLNB carries the risk of false negatives, meaning that the sampled lymph nodes do not contain cancer but other lymph nodes not sampled through the procedure do contain disease. When SLNB was first introduced the false-negative rate was 10%, but over the years it has dropped to 5% in patients offered upfront breast surgery.

The accuracy of SLNB has been questioned following neoadjuvant chemotherapy. Chemotherapy may lead to fibrosis of the lymphatic duct leading to the sentinel lymph node and thus the blue dye or technetium molecule is directed to travel to other lymph nodes that are not the true sentinel nodes. In addition, the order of response of the nodes in the axilla is not known; the sentinel node may respond to treatment and become free of tumor, regardless of whether or not the other axillary nodes still harbor disease. This is of particular concern in patients with clinically node-positive disease who undergo chemotherapy before surgery. However, the sentinel node carries the highest tumor burden and one would argue that if there is no residual disease in the sentinel node then most likely disease has also been cleared from nodes with less tumor burden.

The results of the large multicentre American College of Surgeons Oncology Group ACOSOG-Z1071 trial were reported in 2013 showing a false-negative rate of 12.6% for SLNB after chemotherapy in women initially presenting with N1 axillary disease [20] comparable to the false-negative rate result found from a meta-analysis of studies of SLNB performed after chemotherapy in women who presented with clinically node-negative breast cancer [21]. A smaller study published in 2014 reported a false-negative rate of 5.9% in patients with N1 disease but 38.9% in patients with N2 or N3 disease [22]. The improved accuracy in this recent study may reflect the importance of experience in performing such a procedure after neoadjuvant chemotherapy.

The SENTINA (SENTinel NeoAdjuvant) multicentre study was designed to investigate the value of SLNB before and after neoadjuvant chemotherapy. One of the arms of this study involved patients who presented with clinically node-positive disease (N+) who converted after primary systemic therapy to clinically node-negative disease. After chemotherapy, all women received SLNB followed by ALND. The rate of detecting the sentinel lymph node was 80.1% and the false-negative rate in this arm of the study was 14.2%. It is noteworthy that the false-negative rate was higher for women in this arm who had only 1 sentinel node removed (24.3%) than for those who had 2 sentinel nodes removed (18.5%) [23]. The authors concluded that caution should be used in the use of SLNB after neoadjuvant chemotherapy. Similarly, the Z1071 investigators did not recommend using SLNB instead of ALNB in this setting because the false-negative rate did not achieve their predetermined threshold of 10%.

A recent meta-analysis of 15 studies examining the feasibility and accuracy of SLNB after chemotherapy in clinically node-positive patients reported a false-positive rate of 14% for node-positive patients, which was higher than that for node-negative patients who underwent chemotherapy (4-5%) or node-negative patients who did not undergo chemotherapy (7%). The status of lymph nodes after chemotherapy did not contribute significantly to the heterogeneity of the false-negative rate. The authors concluded that SLNB is feasible after chemotherapy for node-positive breast cancer, but it is not sufficiently accurate to replace ALND [24].

Given the fact that sampling more nodes decreases the likelihood of a false-negative finding, [20, 23] some commentators have suggested that SLNB after chemotherapy is reliable as long as 3 or more nodes are harvested. The choice of technique may also influence the utility of SLNB after systemic therapy. In the Z1071 trial, use of a dual-agent mapping technique with blue dye and radioactive colloid decreased the false-negative rate [20].

The safety of SLNB following neoadjuvant chemotherapy in patients presenting with node-positive breast cancer is currently being assessed with a randomized trial, the NSABP B-51/Radiation Therapy Oncology Group (RTOG) 1304. This trial randomly assigns patients in whom the sentinel lymph nodes have converted to benign nodes following chemotherapy to nodal radiotherapy versus no nodal radiotherapy without further axillary dissection [25].

With the current available literature, it is clear that the identification rate of sentinel lymph node is diminished after NACT as compared to chemotherapy naïve patient. Thus, we advise using dual technique with both blue dye and scintigraphy when performing SLNB after NACT to improve success of the procedure. There is enough evidence to support performing SLNB for patients who are node-negative prior to initiation of chemotherapy. The data is still not decisive regarding SLNB in node-positive patients who converted to node-negative after chemotherapy. The authors of the Z1071 trial concluded that SLNB is reliable when at least 3 sentinel lymph nodes are retrieved, which may not be possible in many patients. Moreover, there is no data on local recurrence in those patients. SLNB after NACT can be offered carefully to selected patients.

## 5. Can SLNB Be Used to Stage the Axilla in Locally Recurrent Breast Cancer following Breast Surgery with or without Prior Axillary Surgery?

It is not clear among the surgical society how to stage the axilla in recurrent breast cancer. Lumpectomy for early breast cancer is associated with a risk of recurrence of roughly 10–15% within 10 years [26, 27]. In patients with locally recurrent breast cancer, axillary staging is important for obtaining locoregional control and determining prognosis. Although SLNB has largely replaced ALNB in the treatment plan for many scenarios in primary breast cancer, its role in recurrent breast cancer has been slower to evolve [28]. A meta-analysis published in 2013 examined repeat SLNB in locally recurrent breast cancer. It included studies with a total of 692 patients in which 43% had had a previous SLNB, 52% a previous ALND, and 4% no previous axillary surgery. The findings indicated that SLNB is technically feasible and accurate for locally recurrent breast cancer [29]. The results of repeat SLNB can also lead to changes in the adjuvant treatment plan [30]. In recurrent breast cancer, lymphatic drainage may be altered, and the drainage of the nodal basin may no longer be via the ipsilateral axilla. Remodeling of lymphatic channels following breast or axillary surgery or radiation therapy can result in drainage to intramammary lymph nodes, the contralateral axilla, or other regional nodal basins. Lymphatic mapping with SLNB in this situation makes it possible to identify regional metastases outside the ipsilateral axilla that conventional imaging techniques and SLNB to the ipsilateral axilla would miss [31]. We advise restaging of the axilla in recurrent breast cancer using SLNB performed by dual technique. This will improve identification rate of the sentinel node and help identify drainage to extra axillary locations.

## 6. Conclusion

The advent of SLNB has caused a dramatic shift away from ALND in the surgical management of the axilla for patients with early breast cancer, resulting in lower complication rates and better quality of life. Findings from major trials are refining the indications for SLNB, but further work is needed to more carefully define the exact populations in whom ALND, with its attendant complications, can safely be avoided. Future trials may examine whether there are scenarios in which it is possible to omit axillary surgery entirely, including SLNB [32].
